# Synergistic anti-obesity effects of *Bifidobacterium breve* BR3 and *Lactiplantibacillus plantarum* LP3 via coordinated regulation of lipid metabolism and gut microbiota

**DOI:** 10.71150/jm.2511001

**Published:** 2025-12-31

**Authors:** Misun Yun, Dooheon Son, Namhee Kim, Se Hee Lee, Eunbee Cho, Sanghyun Lim

**Affiliations:** 1 Advanced Convergence Research Division, World Institute of Kimchi, Gwangju 61755, Republic of Korea; 2 R&D Center, Cell biotech Co. Ltd., Gimpo 10003, Republic of Korea

**Keywords:** obesity, functional probiotics, lipid metabolism, gut microbiota, metabolic regulation

## Abstract

The global rise in obesity and its associated metabolic complications underscores the urgent need for safe and effective interventions. This study investigated the anti-obesity efficacy of a probiotic mixture containing *Bifidobacterium breve* BR3 and *Lactiplantibacillus plantarum* LP3 in C57BL/6 mice with high-fat diet (HFD)-induced obesity. After obesity was established by feeding a 60% kcal HFD, the probiotic mixture was administered orally for 4 weeks. Compared with the control group, mice receiving the *L. plantarum* LP3 and *B. breve* BR3 mixture exhibited significant reductions in body weight and total fat mass, as assessed by Dual-energy X-ray Absorptiometry (DXA) and Echo Magnetic Resonance Imaging (EchoMRI). The probiotic treatment also lowered serum Aspartate Aminotransferase (AST), Alanine Aminotransferase (ALT), and glucose levels, and attenuated lipid accumulation in both hepatic and epididymal adipose tissues. Transcriptomic profiling revealed upregulation of lipolytic genes (*Sirt1*, *Pparα*) and downregulation of lipogenic genes (*Srebp1c*, *Fas*), suggesting that the probiotic mixture promotes lipid catabolism while suppressing lipid synthesis. Additionally, serum adipokine levels were favorably modulated, indicating improved metabolic homeostasis. Gut microbiota analysis demonstrated an increased relative abundance of beneficial genera, including *Akkermansia* and *Bacteroides*, highlighting a microbiome-mediated contribution to the observed metabolic benefits. Overall, our findings indicate that the combined administration of *Lactiplantibacillus plantarum* LP3 and *Bifidobacterium breve* BR3 exerts multi-faceted anti-obesity effects by enhancing lipolysis, regulating lipid metabolism, and restoring a healthy gut microbial balance. This probiotic mixture represents a promising therapeutic approach for managing obesity and related metabolic disorders.

## Introduction

Obesity has become one of the most pressing global health challenges, with its prevalence continuing to rise across both developed and developing countries (Jin et al., 2023; Reinisch et al., 2025; WHO, 2025). The World Health Organization (WHO) estimates that more than 650 million adults are obese, a condition closely linked to metabolic complications such as insulin resistance, type 2 diabetes, non-alcoholic fatty liver disease (NAFLD), and cardiovascular disorders (An et al., 2023; Qin et al., 2012). At the physiological and molecular levels, obesity is characterized by excessive lipid accumulation and dysfunctional adipose remodeling that drive chronic inflammation and metabolic impairment (An et al., 2023; Cho et al., 2023; Konrad and Wueest, 2014; Reinisch et al., 2025; Stefan et al., 2021). The pathogenesis of obesity involves a complex interplay between excessive caloric intake, impaired lipid turnover, and alterations in the gut microbiota (Bäckhed et al., 2004; Fan and Pedersen, 2021; Tilg and Moschen, 2014; Turnbaugh and Gordon, 2009; Turnbaugh et al., 2008). The gut microbiota contributes to host metabolism by regulating energy harvest, bile acid transformation, and production of short-chain fatty acids (Cheng et al., 2022; Clemente et al., 2012; Ejtahed et al., 2020; Yoon et al., 2021). Dysbiosis, defined as the disruption of intestinal microbial homeostasis, has been associated with lipid accumulation, insulin resistance, and chronic metabolic inflammation (Agus et al., 2021; Fan and Pedersen, 2021; Khan et al., 2021). Consequently, modulation of the gut microbiota has emerged as a promising strategy for the management of obesity and related metabolic diseases (Cai et al., 2022; Guo et al., 2024; Kim et al., 2025; Wiciński et al., 2020; Zhao et al., 2018).

Despite the availability of lifestyle interventions and pharmacological treatments, long-term weight management remains challenging, highlighting the need for safe, sustainable, and microbiome-targeted strategies (Soundharrajan et al., 2020; Wiciński et al., 2020). In this context, probiotics have attracted considerable attention as potential modulators of host lipid metabolism, energy expenditure, and inflammatory signaling (Guo et al., 2024; Hou et al., 2025; Kim et al., 2025; Ondee et al., 2025; Turnbaugh et al., 2008). Specific strains of *Lactiplantibacillus* and *Bifidobacterium* have demonstrated anti-obesity potential by reducing body-weight gain, improving serum lipid profiles, and enhancing intestinal barrier function (Cai et al., 2022; Hou et al., 2025; Kobyliak et al., 2016; Ondee et al., 2025). These beneficial effects are often mediated through activation of AMPK dependent lipolytic pathways, suppression of lipogenesis via SREBP-1c and FAS down-regulation, and restoration of gut microbial balance (Ji et al., 2019; Kim et al., 2025; Lee et al., 2025; Soundharrajan et al., 2020). However, the specific mechanisms underlying synergistic interactions among multi-strain probiotics remain incompletely understood (Kim et al., 2025; Ondee et al., 2025). Recent work on next-generation “pharmabiotics” has revealed enhanced metabolic efficacy through coordinated modulation of inflammation, lipid metabolism, and the gut–liver axis (Kim et al., 2025). In this study, we investigated the anti-obesity effects of a probiotic mixture composed of *Lactiplantibacillus plantarum* LP3 and *Bifidobacterium breve* BR3 in a high-fat-diet-induced obese mouse model (Cai et al., 2022; Choi et al., 2024; Guo et al., 2024; Hou et al., 2025; Ondee et al., 2025). Both strains have been previously characterized for safety, stability, and metabolic activity, suggesting their potential as next-generation probiotics for metabolic health improvement (Choi et al., 2024; Ondee et al., 2025). We evaluated their effects on body weight, adiposity, serum lipid metabolism, hepatic and adipose gene expression, and gut microbiota composition (Langin, 2011; Qin et al., 2012). Collectively, our findings demonstrate that this probiotic combination exerts multi-faceted anti-obesity actions by enhancing lipolysis, suppressing lipid accumulation, and restoring gut microbial homeostasis, supporting its promise as a microbiome-targeted approach for combating obesity and metabolic dysfunction.

## Materials and Methods

### Probiotic supplementation (BR3 and LP3)

The probiotic preparation was produced by Cell Biotech (Republic of Korea) as a mixed microtablet formulation containing *Bifidobacterium breve* CBT BR3 (KCTC 12201BP) and *Lactiplantibacillus plantarum* CBT LP3 (KCTC 10782BP). Each daily dose provided a total of 2 × 10⁸ CFU of the two strains in a 1:1 ratio, suspended in 200 µl of Dulbecco’s Phosphate-Buffered Saline (DPBS) for oral gavage, unless otherwise specified (Choi et al., 2024).

### Animal model and diet

Male C57BL/6 mice (6 weeks old) were purchased from OrientBio Co. (Korea). All animal procedures were approved by the Institutional Animal Care and Use Committee of the World Institute of Kimchi IACUC (WIKIM IACUC 202330) in accordance with institutional and national guidelines. After a 1- week acclimation period, mice were randomly assigned to three groups: NCD (normal chow diet; n = 9; vehicle DPBS), HFD (high-fat diet; n = 7; 60% kcal from fat), BRLP (HFD + probiotic mixture; n = 8). Mice in HFD and BRLP groups were fed the for 8 weeks to induce obesity. Subsequently, the BRLP groups received the probiotic mixture daily for 4 weeks, while HFD mice received vehicle alone. Body weight was recorded weekly, and food intake was recorded three times per week.

### Body composition analysis

Body composition was assessed using dual-energy X-ray absorptiometry (DXA; InAlyzer, Medikors, Korea) and Echo magnetic resonance imaging (EchoMRI-500, EchoMRI, USA). DXA color-mapped images were acquired to qualitatively visualize the distribution of body fat and lean mass.

### Serum biochemical analysis

Serum was separated by centrifugation of whole blood at 3,000 rpm for 10 min. Biochemical parameters were subsequently analyzed using a Fujifilm DRI-CHEM 7000i automated analyzer to determine the concentrations of aspartate aminotransferase (AST), alanine aminotransferase (ALT), total cholesterol (TCHO), and glucose (GLU).

### Histological analysis

Liver and epididymal adipose tissues were preserved in 10% neutral-buffered formalin, processed through paraffin embedding, and sliced into 5-µm sections. The tissue sections were stained with hematoxylin and eosin (H&E) to examine general histomorphology and with Oil Red O to visualize lipid deposition. Images were obtained using an Olympus BX53 light microscope, and quantitative analyses were performed in ImageJ software. Adipocyte cross-sectional area and hepatic lipid accumulation were calculated from at least five randomly selected microscopic fields per animal.

### Quantitative real-time PCR analysis of gene expression

Total RNA from liver and epididymal fat tissues was extracted using the Hybrid-R^TM^ (GeneAll Biotechnology Co., Ltd., Korea). A fixed amount of 500 ng of RNA was reverse-transcribed into complementary DNA (cDNA) using the TOPscript^TM^ cDNA Synthesis Kit (Enzynomics, Korea). Quantitative real-time PCR (qPCR) was performed to analyze the expression of target genes in liver and adipose tissues. Each reaction contained 200 ng of cDNA, 10 µl of TOPreal^TM^ SYBR Green qPCR PreMIX (Enzynomics, Korea), 3 µl of RNAse-free distilled water, and 2 µl of a primer mixture specific to each target gene. Amplification was carried out on a CFX96 Real-Time PCR

Detection System (Bio-Rad, USA) under the following cycling conditions: initial denaturation at 98℃ for 15 min, followed by 39 cycles at 95℃ for 10 s, 57℃ for 15 s, and 72℃ for 15 s, with a subsequent melting-curve analysis to confirm product specificity. Glyceraldehyde-3-phosphate dehydrogenase (GAPDH) was used as the housekeeping gene for normalization of gene expression levels.

### Bioinformatics pipelines for microbiota analyses

Paired-end raw sequencing data were imported into QIIME2 (version 2023.2) as artifacts, and demultiplexed using the QIIME2 plugin (Jo et al., 2024). Low-quality reads were filtered, and denoised paired-end sequences merged using the DADA2 algorithm to generated high-confidence amplicon sequence variants (ASVs). After quality control, alpha- and beta-diversity analyses were performed through the q2-diversity plugins. Alpha diversity was calculated using observed ASVs, Faith’s Phylogenetic Diversity (Faith’s PD), and Shannon index and Pielou’s evenness. A rarefraction table was constructed by subsampling features at multiple sequencing depths. Beta-diversity was assessed using Weighted UniFrac, Unweighted UniFrac, Bray–Curtis, and Jaccard distances, and principal coordinate analysis (PCoA) plots were visualized with Emperor. Taxonomic assignment of ASVs was performed using a Naïve Bayes classifier trained on the SILVA-138.1 database via q2-feature-classifier plugin.

### Taxonomic composition analyses

Stacked box plots were generated to visualize the relative abundance of microbial taxonomic level (Phylum, Family, Genus, and Species). Sequences that could not be classified at a given taxonomic level but were identified at higher level were grouped under the category “unclassified (_uc).” Differentially abundant taxa were identified using the linear discriminant analysis (LDA) effect size (LEfSe) method, applying an LDA score threshold of 2.0 and statistical significance determined by the Kruskal–Wallis test followed by the Wilcoxon test (*p* < 0.05). The LEfSe analysis was conducted through the Galaxy platform available online at http://galaxy.biobakery.org/ (Segata et al., 2011).

### Data availability statement

Publicly available sequencing dataset can be found in the NCBI BioProject under accession number PRJNA1344464.

### Predicted functional profiling (PICRUSt2)

Predicted metagenomic functions were inferred from the 16S rRNA sequencing data using PICRUSt2 (version 2.0). The generated functional profiles were annotated against the MetaCyc database to identify associated metabolic pathways and grouped into Superclass categories. Comparative analyses of pathway abundances and their relationships with host metabolic indices were subsequently performed.

### Statistical analyses

All graphical visualizations were generated using GraphPad Prism version 7.05 (GraphPad Inc., USA) and OriginPro^®^ 2024 (OriginLab Corporation, USA). Statistical evaluations of alpha and beta diversity were conducted using QIIME2 plugins (*q2-alpha-group-significance* and *q2-beta-group-significance*). Alpha diversity was compared using a one-tailed Student’s *t*-test, whereas beta-diversity differences were determined through permutation multivariate analysis of variance (PERMANOVA) with 999 permutations. At the genus and species levels, relative abundance data were normalized using the built-in normalization function in GraphPad Prism, and intergroup comparisons were performed with Tukey’s multiple comparison test. Correlations between the relative abundances of selected taxa and metabolic parameters were assessed using a one-tailed Spearman’s rank correlation test with adjusted *p*-values. Data are presented as Mean ± standard deviation (SD), and statistical significance was defined as *p* < 0.05.

## Results

### Probiotic mixture BRLP reduces HFD-induced weight gain without altering food intake

**Fig. 1.**
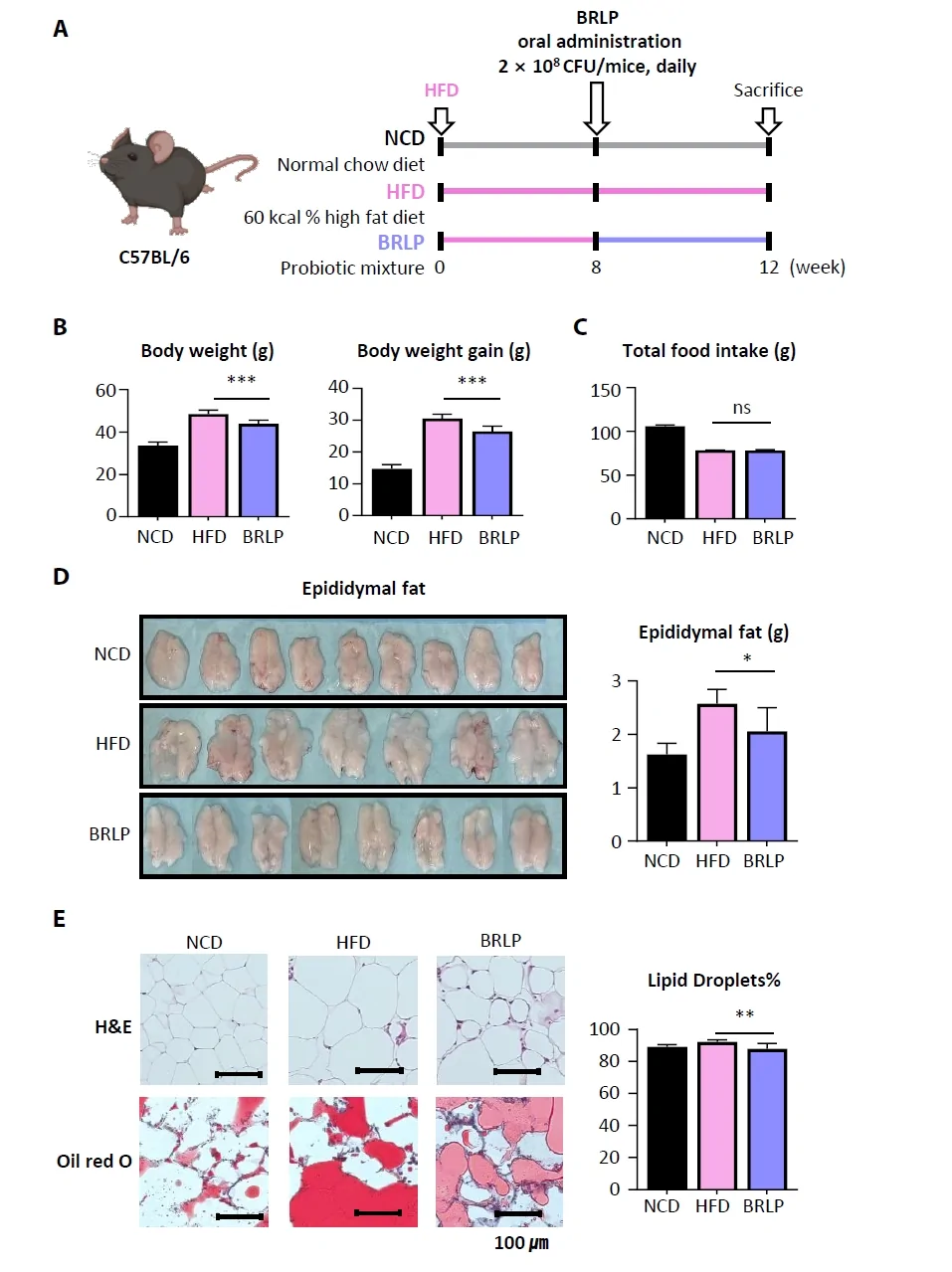
Administration of BRLP alleviates high-fat-diet–induced obesity in mice. (A) Experimental design of the high-fat diet (HFD; 60% kcal fat)–induced obesity model. Male C57BL/6 mice were fed a normal chow diet (NCD), an HFD, or an HFD supplemented with the mixed probiotics *Bifidobacterium breve* CBT BR3 + *Lactiplantibacillus plantarum* CBT LP3 (BRLP). (B, C) BRLP administration significantly reduced body weight and cumulative weight gain compared with HFD controls during the 12-week feeding period. (D) Total food intake did not differ between HFD and BRLP groups, indicating a metabolic rather than anorectic effect. (E) Representative gross images of epididymal fat depots showing visibly smaller fat pads in BRLP mice. Tissue weight was significantly lower in BRLP than in HFD controls. Hematoxylin–eosin (H&E) and Oil Red O staining of epididymal adipose tissue showing decreased lipid accumulation and smaller adipocytes in the BRLP group. Data are presented as Mean ± SD. Statistical significance was determined by one-way ANOVA followed by Tukey’s post hoc test (*p* < 0.05, ^*^p < 0.01, ^**^p < 0.001).

High-fat diet (HFD) feeding produced progressive body-weight gain compared with the normal-chow diet (NCD) group. Oral administration of the probiotic mixture BRLP (*Bifidobacterium breve* BR3 + *Lactiplantibacillus plantarum* LP3) significantly attenuated both body weight and cumulative weight gain (*p* < 0.001) without affecting total food intake, suggesting a metabolic rather than anorectic mechanism (Fig. 1A–1E). Epididymal fat-pad weight was markedly lower in BRLP-treated mice (*p* < 0.05–0.01), and histological evaluation revealed smaller adipocytes and reduced lipid droplet density by H&E and Oil Red O staining (Fig. 1D–1E). Quantification confirmed a significant decrease in adipocyte cross-sectional area (*p* < 0.001). Consistently, liver weight was reduced and hepatic steatosis alleviated in the BRLP group relative to HFD controls (Fig. S1A–S1B), indicating systemic mitigation of lipid overload.

### Body composition and serum chemistry are improved by BRLP

**Fig. 2.**
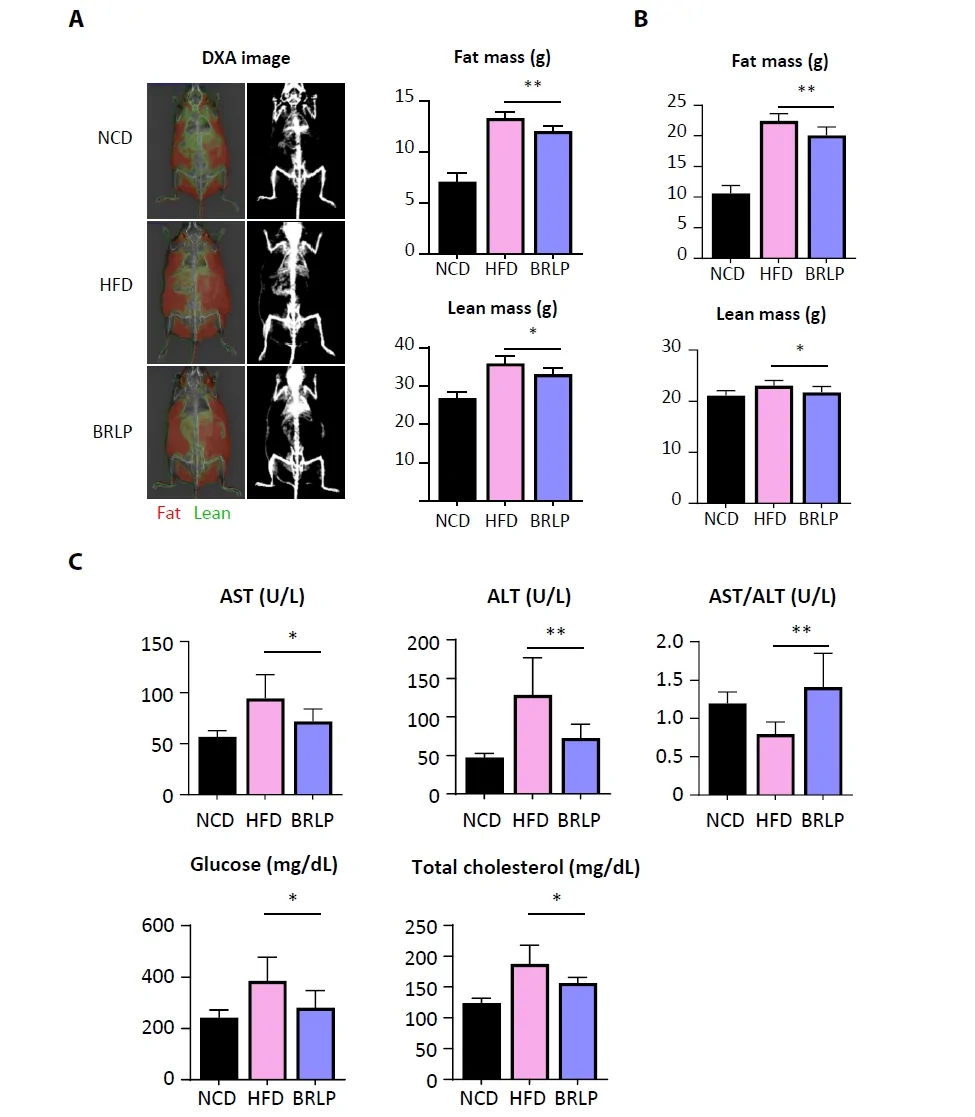
BRLP reduces obesity-related body composition and serum biochemical markers in high-fat-diet–induced obese mice. (A) Representative dual-energy X-ray absorptiometry (DXA) scan images showing total body composition in each group (NCD, HFD, and BRLP). Quantitative analysis of fat and lean mass measured by DXA demonstrated a significant reduction in total fat mass and improved lean-to-fat ratio in the BRLP group compared with HFD controls. (B) EchoMRI-based measurements confirmed decreases in fat and lean mass in BRLP-treated mice relative to the HFD group. (C) Serum biochemical parameters, including glucose (GLU), total cholesterol (TCHO), aspartate aminotransferase (AST), and alanine aminotransferase (ALT), were significantly improved in BRLP mice compared with HFD controls, indicating amelioration of metabolic dysfunction. Data are presented as Mean ± SD. Statistical significance was determined by one-way ANOVA with Tukey’s post hoc test (*p* < 0.05, ^*^p < 0.01, ^**^p < 0.001).

DXA and EchoMRI analyses demonstrated substantially lower total fat mass in BRLP compared with HFD, while the lean-to-fat mass ratio improved, indicating a selective reduction in adiposity rather than lean-mass loss (Fig. 2A–2B). Biochemical analysis revealed that BRLP significantly decreased serum glucose and total cholesterol and attenuated AST/ALT elevations associated with hepatic stress (*p* < 0.05–0.01; Fig. 2C). These findings indicate that probiotic supplementation restores systemic metabolic balance disrupted by HFD.

### BRLP reshapes gut microbial diversity and reinforces intestinal barrier function

**Fig. 3.**
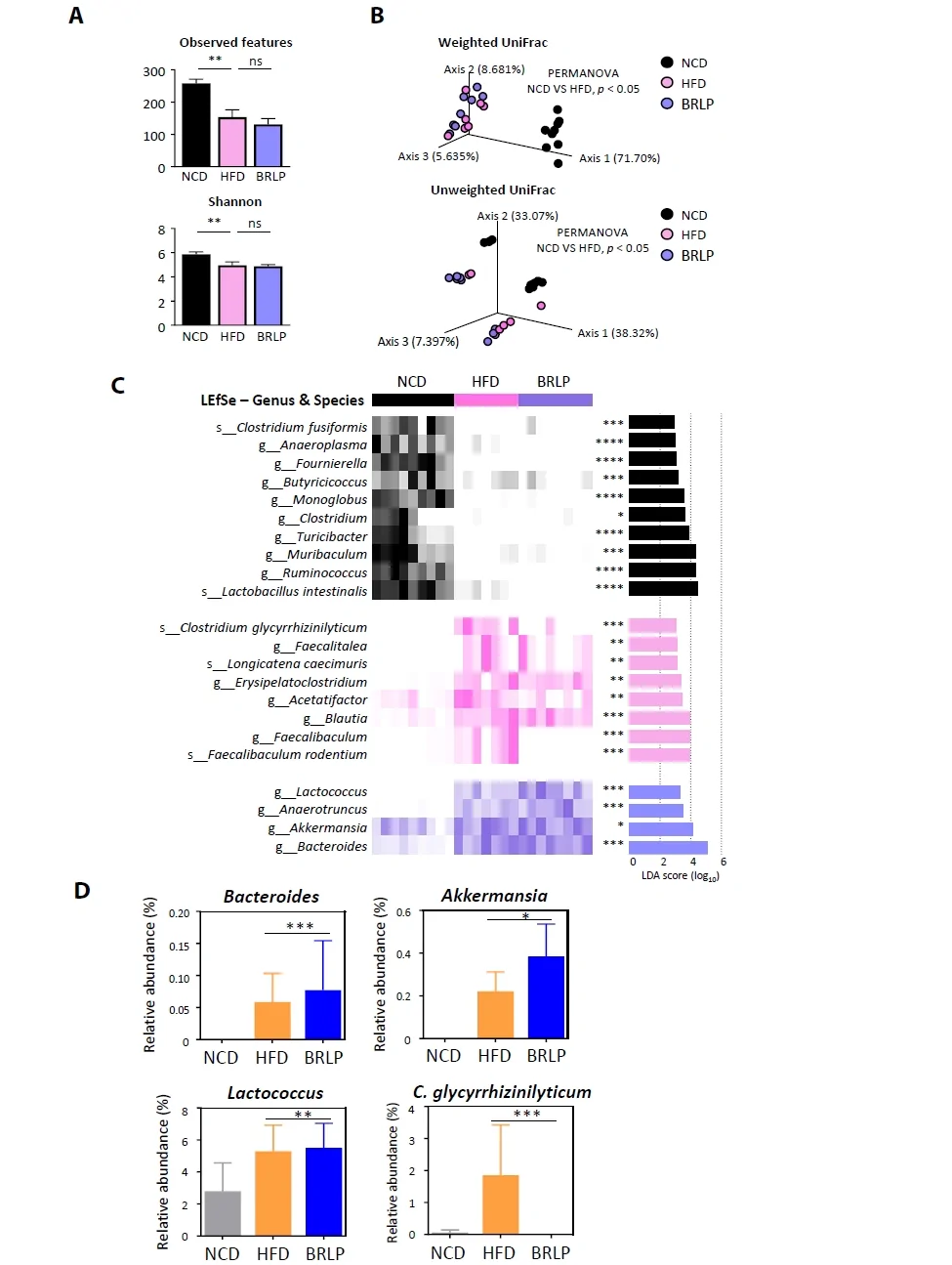
BRLP administration alters gut microbial diversity and taxonomic composition in high-fat-diet–induced obese mice. (A) Alpha-diversity indices (Observed features and Shannon index) of fecal microbiota were significantly reduced in HFD mice compared with NCD controls, whereas BRLP supplementation partially restored microbial diversity. Data are presented as Mean ± SD (^**^p < 0.01). (B) Principal coordinate analysis (PCoA) plots based on Weighted and Unweighted UniFrac distances showing distinct clustering of microbial communities among NCD, HFD, and BRLP groups. PERMANOVA confirmed significant compositional dissimilarities between groups (*p* < 0.05). (C) Heatmap and linear discriminant analysis (LDA) effect size (LEfSe) results illustrating differential taxa abundance at the genus (g) and species (s) levels across groups. Bars represent log₁₀ LDA scores, highlighting taxa enriched in each condition. Statistical significance: *p* < 0.05, ^*^p < 0.01, ^**^p < 0.001, ^***^p < 0.0001.

Alpha-diversity indices (Observed ASVs, Faith’s PD, Shannon, Pielou) declined under HFD but increased toward baseline following BRLP treatment. Principal-coordinate analysis (PCoA) based on Weighted and Unweighted UniFrac distances demonstrated distinct clustering of BRLP samples apart from HFD, confirmed by PERMANOVA (*p* < 0.01) (Fig. 3A–3B). Parallel taxonomic bar plots at the phylum, family, and genus levels showed clear microbiota remodeling across diet groups, with BRLP reversing several HFD-associated compositional distortions (Fig. S2A–S2C). LEfSe analysis revealed enrichment of beneficial genera such as *Akkermansia*, *Bacteroides*, and *Lactococcus* in BRLP, accompanied by depletion of *Clostridium glycyrrhizinilyticum*, a taxon associated with lipid accumulation (LDA > 2.0, *p < 0.05–0.0001; Fig. 3C). At the host level, intestinal-tissue qPCR analyses demonstrated reduced pro-inflammatory cytokine expression and up-regulation of anti-inflammatory and tight-junction genes (*Ocln*, *Cldn*, *Tjp*), supporting improved epithelial integrity (*p* < 0.05–0.001; Fig. S3A–S3D). Together, these data indicate that BRLP corrects HFD-induced dysbiosis and strengthens the gut barrier, potentially reducing endotoxin leakage and chronic low-grade inflammation.

### Functional pathway prediction links microbial shifts to host lipid metabolism

**Fig. 4.**
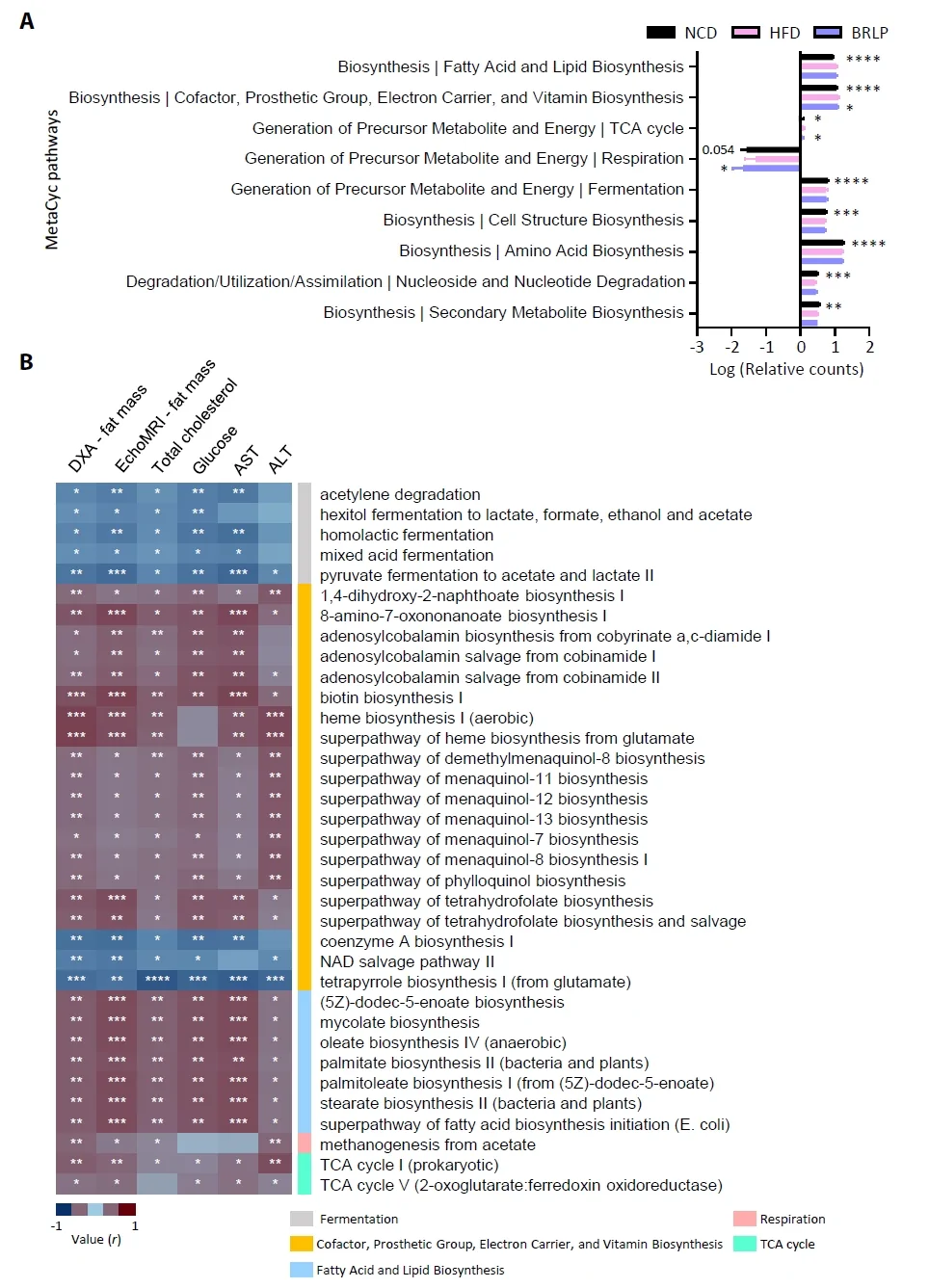
Correlation between anti-obesity efficacy of BRLP and predicted microbial metabolic pathways. (A) Forest plot showing functional pathways significantly altered among NCD, HFD, and BRLP groups as predicted by PICRUSt2 analysis at the MetaCyc Superclass level. Pathways are represented by log₁₀transformed relative abundance values. BRLP administration increased pathways related to NAD^⁺^ regeneration and CoA biosynthesis, while reducing those associated with fatty acid and TCA cycle metabolism. (B) Heatmap depicting Spearman’s correlation between significantly changed MetaCyc Superclass pathways and obesity-related biomarkers, including body weight, fat mass, serum glucose, total cholesterol, and transaminase levels. Blue and red indicate negative and positive correlations, respectively. Statistical significance: *p* < 0.05, ^*^p < 0.01, ^**^p < 0.001, ^***^p < 0.0001.

PICRUSt2-based functional inference revealed broad metabolic remodeling of the microbial community. Compared with HFD, BRLP reduced the relative abundance of pathways involved in the TCA cycle and fatty-acid/lipid biosynthesis, while enhancing respiration-related energy pathways (Fig. 4A). Correlation mapping between microbial functional pathways and host metabolic indices showed that TCA and lipid-biosynthetic pathways correlated positively with fat mass, serum TCHO, GLU, AST, and ALT, whereas fermentation pathways linked to short-chain-fatty-acid (SCFA) production correlated negatively (Fig. 4B). Moreover, pathways for NAD salvage II, coenzyme A biosynthesis I, and tetrapyrrole (heme) biosynthesis I were negatively associated with obesity parameters (*p* < 0.05–0.0001), implying enhanced NAD^⁺^ regeneration, CoA availability, and mitochondrial oxidative capacity biochemical contexts that favor SIRT1-driven lipid catabolism and fatty-acid oxidation.

### BRLP rewires adipose gene networks to favor lipolysis over lipogenesis

**Fig. 5.**
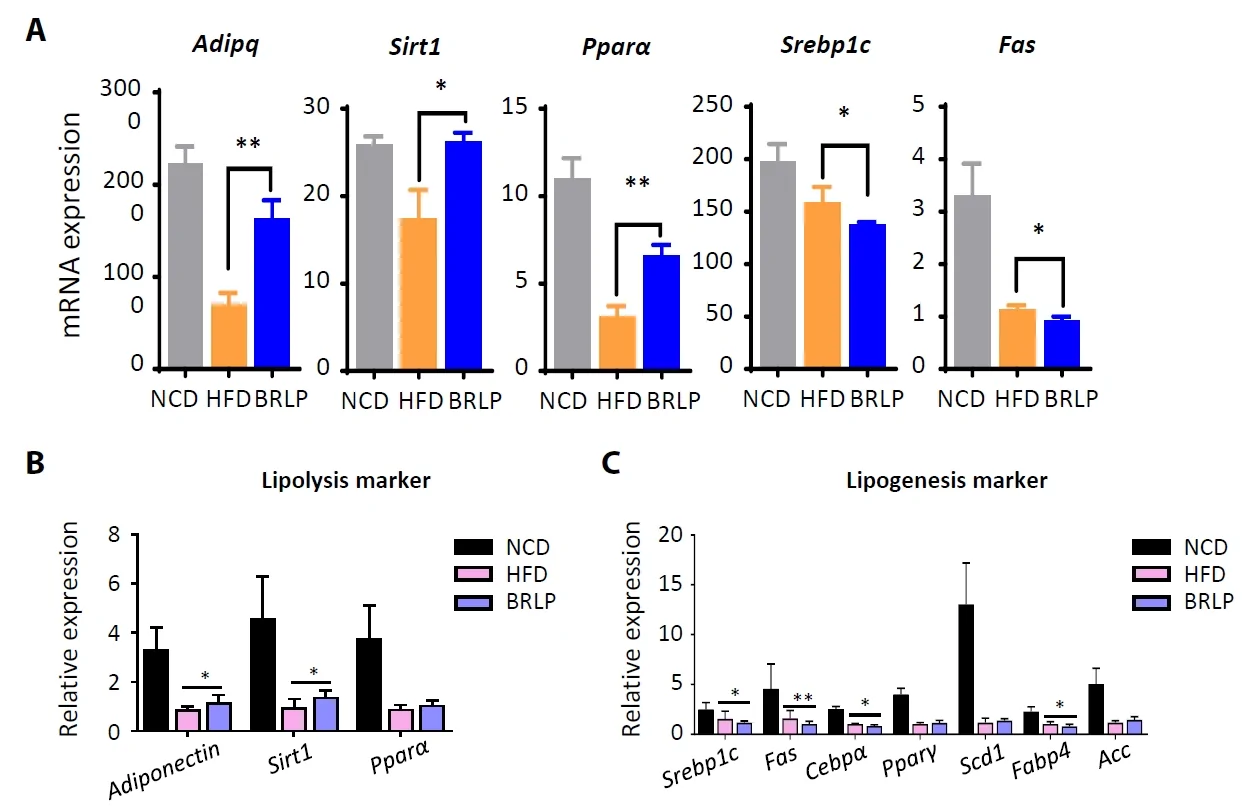
BRLP supplementation promotes lipolysis and suppresses lipogenesis in epididymal adipose tissue of obese mice. (A) RNA-seq analysis of epididymal adipose tissue showing transcriptional remodeling of lipid metabolism pathways following BRLP administration. (B) Box plots showing relative mRNA expression of lipolytic genes (*Sirt1*, *Pparα*) in epididymal fat, quantified by qRT-PCR using the 2^⁻ΔΔCT^ method. Sirt1 (histone deacetylase sirtuin 1) was significantly upregulated in BRLP-treated mice, indicating enhanced fatty acid oxidation. (C) Box plots showing relative mRNA expression of lipogenic genes (*Srebp1c*, *Fas*, *Cebpα*, *Pparγ*, *Scd1*, *Fabp4*, and *Acc*) in epididymal fat. BRLP supplementation significantly downregulated these genes involved in de novo lipogenesis and adipocyte differentiation, supporting the observed reduction in adiposity. Data are presented as Mean ± SD. Statistical significance was determined by one-way ANOVA followed by Tukey’s post hoc test (*p* < 0.05, ^*^p < 0.01, ^**^p < 0.001).

Adipose-tissue transcriptomic and qRT-PCR analyses showed marked up-regulation of lipolytic genes (*Sirt1*, *Pparα*) and concurrent down-regulation of lipogenic and adipogenic genes (*Srebp1c*, *Fas*) (Fig. 5A–5C). These molecular alterations coincided with smaller adipocytes and reduced hepatic lipid accumulation (Fig. S1A–S1B), indicating that BRLP promotes fatty-acid oxidation and suppresses de novo lipogenesis. The simultaneous up-regulation of Adipoq further suggests improved adipokine-mediated lipid turnover and insulin sensitivity, supporting whole-body metabolic restoration. Collectively, integration of the molecular, histological, and microbial data (Figs. 1–5, S1–S3) demonstrates that BRLP exerts coordinated actions along the gut–liver–adipose axis, leading to reduced adiposity and improved metabolic homeostasis.

## Discussion

**Fig. 6.**
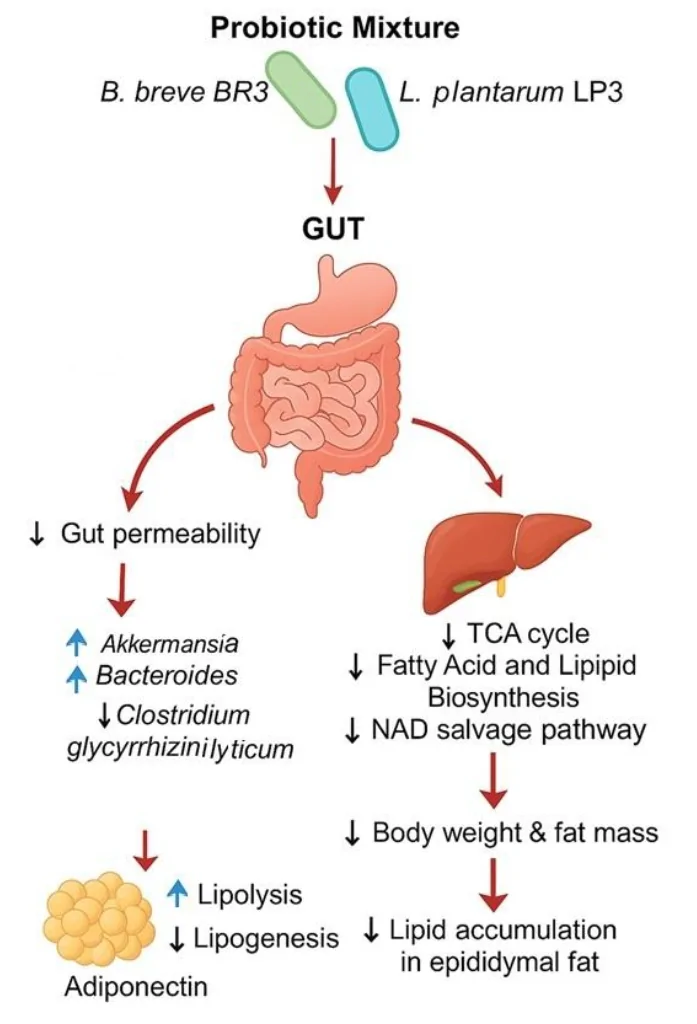
Proposed mechanism of BRLP-mediated anti-obesity effects integrating host lipid metabolism and gut microbiota modulation.

This study demonstrates that oral administration of a two-strain probiotic mixture comprising *Bifidobacterium breve* BR3 and *Lactiplantibacillus plantarum* LP3 and produces multifaceted antiobesity effects in HFD-induced obese mice. BRLP supplementation significantly reduced body weight, fat mass, and hepatic lipid accumulation, accompanied by biochemical, molecular, and microbial remodeling indicative of restored metabolic equilibrium. Mechanistically, BRLP activated lipolytic and β-oxidation pathways (*Sirt1*, *Pparα*) while repressing lipogenesis and adipogenesis (*Srebp1c*, *Fas*), leading to smaller adipocytes and reduced hepatic steatosis. These transcriptional reprogramming events are consistent with earlier findings showing that probiotic strains of *Lactobacillus* and *Bifidobacterium* promote lipid catabolism through *Sirt1*-*Pparα* and AMPK signaling cascades (Everard et al., 2013; Turnbaugh et al., 2008). In addition, Up-regulation of *Adipoq* suggests enhanced adiponectin-mediated insulin sensitivity and fatty-acid clearance, which further contributes to metabolic improvement. BRLP supplementation also reshaped the gut microbiota composition, enriching beneficial taxa such as *Akkermansia* and *Bacteroides*, which are known to support mucosal barrier integrity, stimulate SCFA production, and facilitate fatty-acid oxidation (Everard et al., 2013). Concurrent up-regulation of tight-junction genes indicates improved epithelial barrier function, supporting the hypothesis that BRLP mediates its effects partly through microbiome–barrier–metabolism cross-talk. Functional predictions showed decreased microbial capacities for lipid and TCA cycle metabolism, while enhancing NAD^⁺^, CoA, and heme biosynthetic pathways, collectively favoring oxidative lipid catabolism and mitochondrial respiration. These multi-layered host microbe interactions convergence on a model in which BRLP mitigates obesity by rewiring lipid metabolism, strengthening intestinal barrier integrity, and modulating gut microbial functionality. A schematic representation of this integrated mechanism is illustrated in Fig. 6, which summarizes the proposed *Sirt1*-*Pparα* activation and *Srebp1c*-*Fas* suppression pathways within the host-microbiome framework. Importantly, this defined probiotic formulation holds translational potential for human health. The constituent strains, *B. breve* and *L. plantarum*, have both been granted QPS (Qualified Presumption of Safety) status by the European Food Safety Authority and GRAS (Generally Recognized As Safe) designation by the U.S. FDA, confirming their suitability for human consumption (Sanders et al., 2019). Based on interspecies dose conversion, the effective mouse dose of 2 × 10⁸ CFU/day corresponds to an approximate human-equivalent dose of ~1 × 10⁹ CFU/day, well within the safe and efficacious range used in commercial functional probiotic preparations (Hill et al., 2014; Valdes et al., 2018). Therefore, the BRLP formulation has practical potential as a microbiome-based functional food or therapeutic supplement for the prevention and management of obesity and related metabolic disorders. Collectively, this study expands current understanding of how probiotics influence systemic energy homeostasis through both host and microbial pathways. By integrating molecular, biochemical, and metagenomic evidence, our findings highlight BRLP as a promising and mechanistically defined probiotic candidate for next-generation microbiome-targeted interventions against metabolic syndrome.

## Supplementary Information

Table S1. Spearman correlation (r-value) between functional pathways and obesity biomarkers

Table S2. Spearman correlation (*p*-value) between functional pathways and obesity biomarkers

Table S3. List of primers used in present study

Fig. S1. (A) The representative images and weight of liver tissue of experimental animal models. Significantly decreased liver weight in the BRLP group compared to the HFD group. (B) H&E and Oil red O staining of liver tissue.

Fig. S2. The stacked box plots of taxonomic composition at the (A) phylum, (B) family, and (C) genus level.

Fig. S3. Modulation of intestinal inflammatory and barrier-related gene expression with BRLP administration. The relative gene expression of (A) pro-inflammatory and (B) anti-inflammatory markers in the small intestine and large intestine.
