# Supplementary material for: Synergistic anti-obesity effects of Bifidobacterium breve BR3 and Lactiplantibacillus plantarum LP3 via coordinated regulation of lipid metabolism and gut microbiota
Source: J Microbiol. 2025 Dec 31;63(12):e2511001. doi: 10.71150/jm.2511001 (PMC13577009; doi:10.71150/jm.2511001)
Supplement: Supplementary file 1 [file jm-2511001-Supplementary-Fig-S1.pdf]

Fig. S1

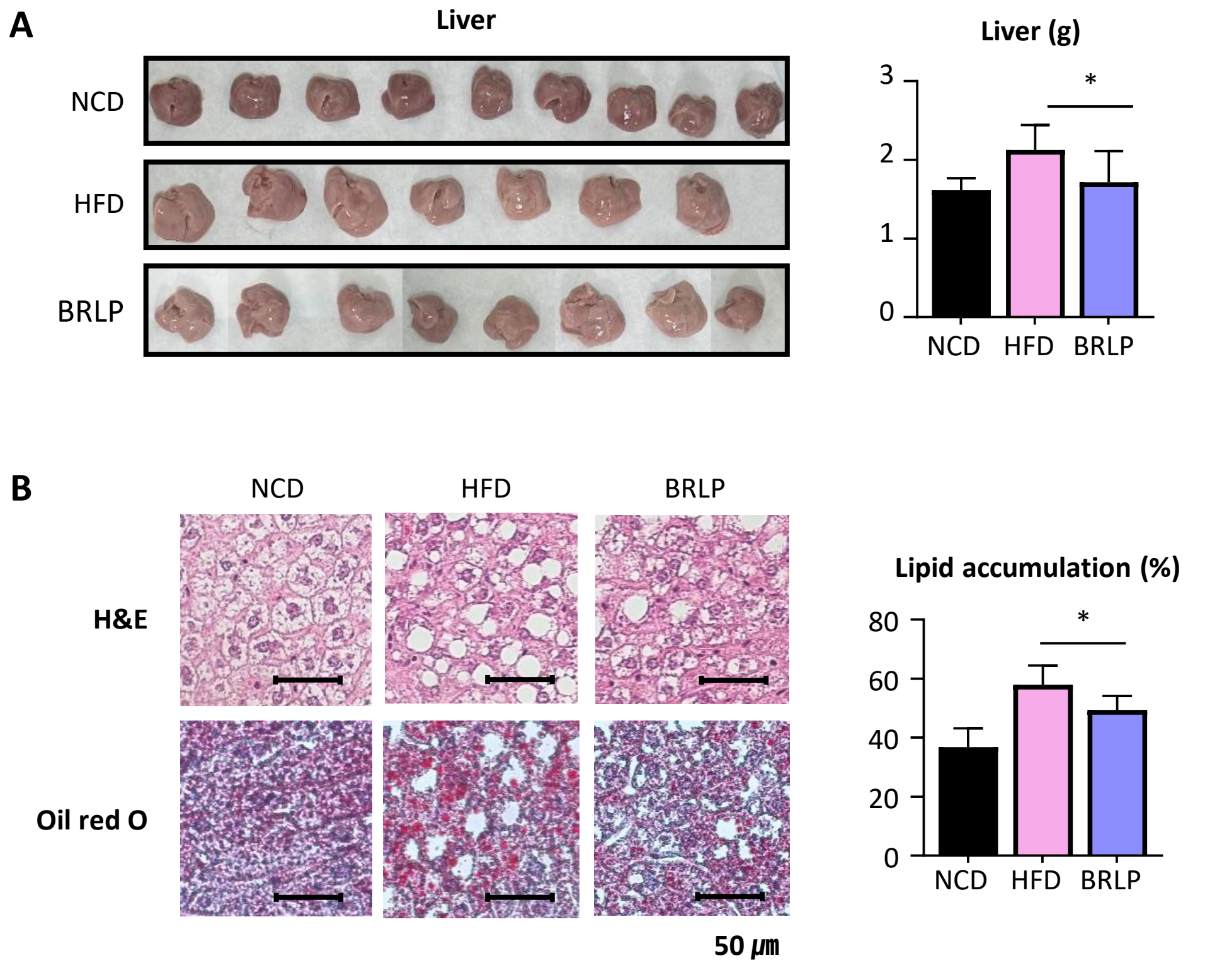

Fig. S1. (A) The representative images and weight of liver tissue of experimental animal models. Significantly decreased liver weight in the BRLP group compared to the HFD group. (B) H&E and Oil red O staining of liver tissue. Decreased lipid accumulation in liver tissue in the BRLP group compared to the HFD group. Data are presented as Mean ± SD. * $p < 0.05$.
