# Supplementary material for: Synergistic anti-obesity effects of Bifidobacterium breve BR3 and Lactiplantibacillus plantarum LP3 via coordinated regulation of lipid metabolism and gut microbiota
Source: J Microbiol. 2025 Dec 31;63(12):e2511001. doi: 10.71150/jm.2511001 (PMC13577009; doi:10.71150/jm.2511001)
Supplement: Supplementary file 2 [file jm-2511001-Supplementary-Fig-S2.pdf]

Fig. S2

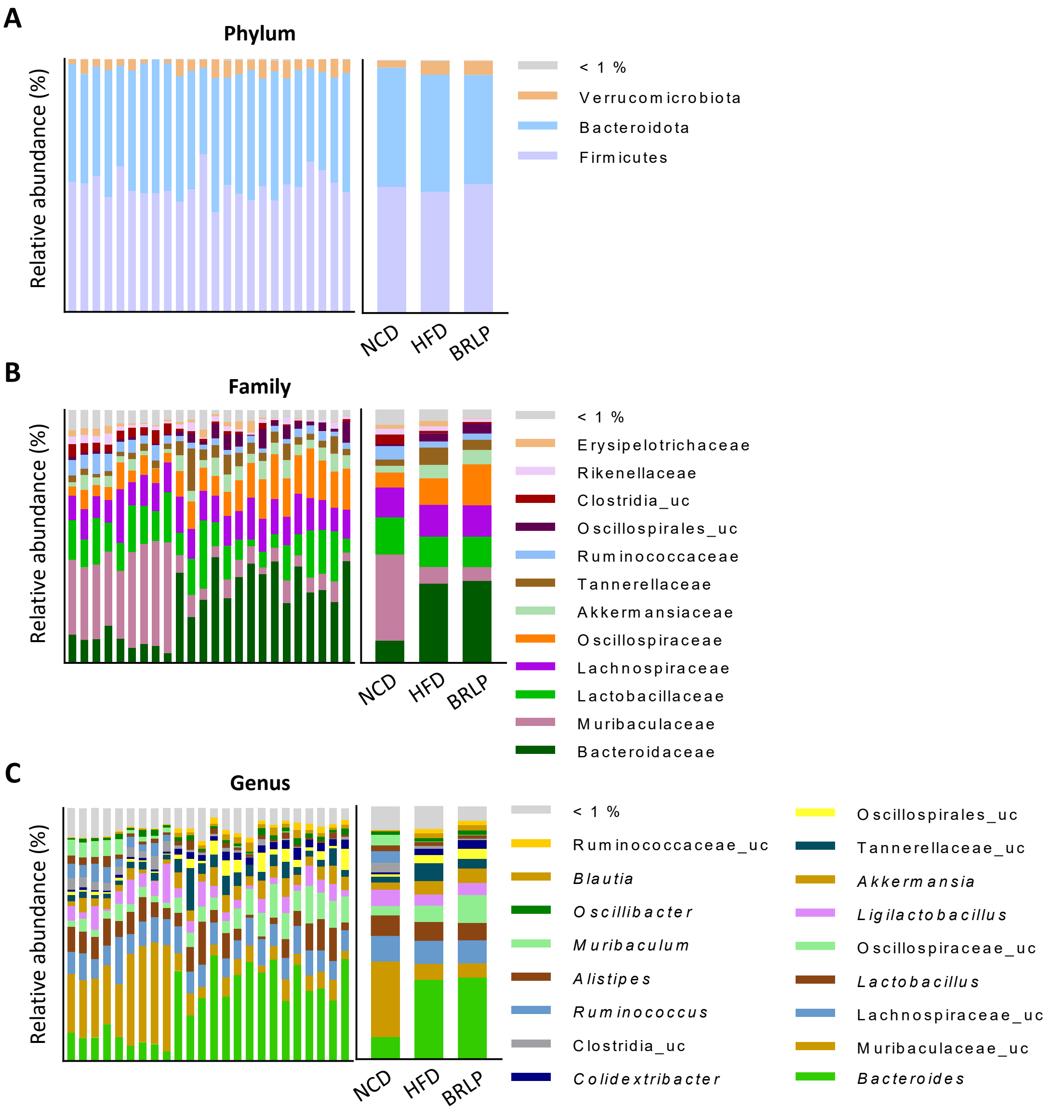

Fig. S2. The stacked box plots of taxonomic composition at the (A) phylum, (B) family, and (C) genus level. Data are presented as mean of relative abundance.
