# Supplementary material for: Synergistic anti-obesity effects of Bifidobacterium breve BR3 and Lactiplantibacillus plantarum LP3 via coordinated regulation of lipid metabolism and gut microbiota
Source: J Microbiol. 2025 Dec 31;63(12):e2511001. doi: 10.71150/jm.2511001 (PMC13577009; doi:10.71150/jm.2511001)
Supplement: Supplementary file 3 [file jm-2511001-Supplementary-Fig-S3.pdf]

Fig. S3

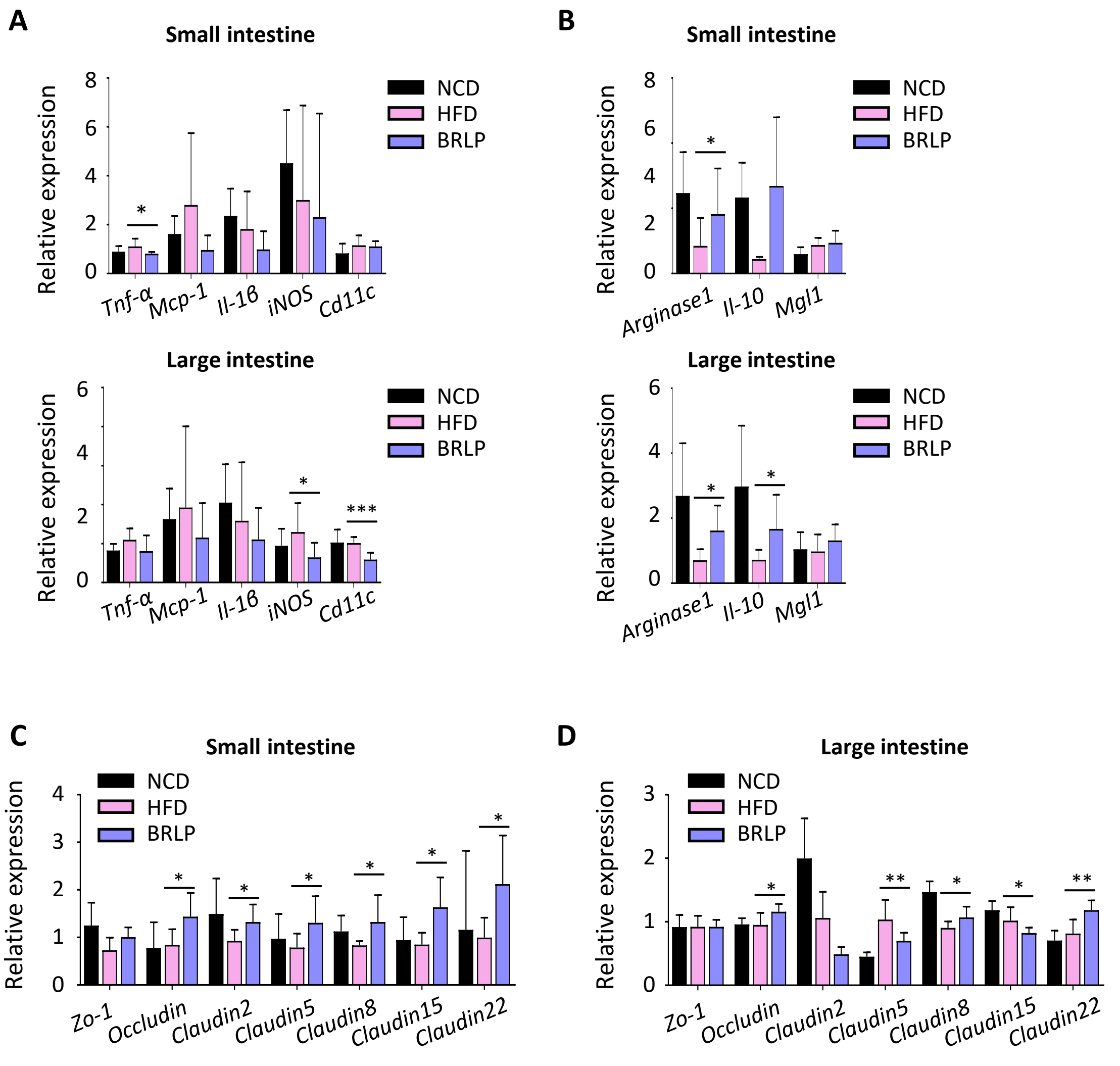

Fig. S3. Modulation of intestinal inflammatory and barrier-related gene expression with BRLP administration. The relative gene expression of (A) pro-inflammatory and (B) anti-inflammatory markers in the small intestine and large intestine. Administration of BRLP increased the expression of tight junction genes in the (C) small intestine and (D) large intestine. Data are presented as Mean ± SD. * $p < 0.05$, ** $p < 0.01$, *** $p < 0.001$.
