# Supplementary material for: Synergistic anti-obesity effects of Bifidobacterium breve BR3 and Lactiplantibacillus plantarum LP3 via coordinated regulation of lipid metabolism and gut microbiota
Source: J Microbiol. 2025 Dec 31;63(12):e2511001. doi: 10.71150/jm.2511001 (PMC13577009; doi:10.71150/jm.2511001)
Supplement: Supplementary file 4 [file jm-2511001-Supplementary-Table-S1.pdf]

Table S1. Spearman correlation (*r*-value) between functional pathways and obesity biomarkers

Superclass	Functional pathway	*r*-value					
		DXA – fat mass	EchoMRI – fat mass	Total Cholesterol (mg/dL)	Glucose (mg/dL)	AST (U/L)	ALT (U/L)
Fermentation	acetylene degradation	-0.438	-0.495	-0.375	-0.482	-0.530	-0.290
	hexitol fermentation to lactate, formate, ethanol and acetate	-0.386	-0.451	-0.411	-0.481	-0.330	-0.200
	homolactic fermentation	-0.452	-0.526	-0.397	-0.545	-0.582	-0.346
	mixed acid fermentation	-0.391	-0.423	-0.355	-0.466	-0.480	-0.248
	pyruvate fermentation to acetate and lactate II	-0.553	-0.612	-0.435	-0.562	-0.630	-0.432
Cofactor, Prosthetic Group, Electron Carrier, and Vitamin Biosynthesis	1,4-dihydroxy-2-naphthoate biosynthesis I	0.482	0.429	0.442	0.495	0.416	0.564
	8-amino-7-oxononanoate biosynthesis I	0.583	0.654	0.541	0.583	0.654	0.472
	adenosylcobalamin biosynthesis from cobyrinate a,c-diamide I	0.467	0.532	0.464	0.567	0.589	0.348
	adenosylcobalamin salvage from cobinamide I	0.462	0.525	0.444	0.551	0.574	0.335
	adenosylcobalamin salvage from cobinamide II	0.481	0.546	0.480	0.581	0.604	0.370
	biotin biosynthesis I	0.606	0.671	0.543	0.592	0.658	0.485
	heme biosynthesis I (aerobic)	0.675	0.610	0.538	0.325	0.563	0.644
	superpathway of heme biosynthesis from glutamate	0.685	0.622	0.524	0.326	0.564	0.640
	superpathway of demethylmenaquinol-8 biosynthesis	0.479	0.424	0.445	0.496	0.416	0.564
	superpathway of menaquinol-11 biosynthesis	0.476	0.415	0.445	0.486	0.391	0.574
	superpathway of menaquinol-12 biosynthesis	0.476	0.415	0.445	0.486	0.391	0.574
	superpathway of menaquinol-13 biosynthesis	0.476	0.415	0.445	0.486	0.391	0.574
	superpathway of menaquinol-7 biosynthesis	0.455	0.389	0.421	0.466	0.377	0.558
	superpathway of menaquinol-8 biosynthesis I	0.477	0.417	0.444	0.472	0.380	0.576
	superpathway of phylloquinol biosynthesis	0.482	0.429	0.442	0.495	0.416	0.564
	superpathway of tetrahydrofolate biosynthesis	0.565	0.622	0.427	0.569	0.560	0.408
	superpathway of tetrahydrofolate biosynthesis and salvage	0.537	0.597	0.414	0.557	0.544	0.386
	coenzyme A biosynthesis I	-0.574	-0.596	-0.461	-0.572	-0.564	-0.358
	NAD salvage pathway II	-0.498	-0.525	-0.426	-0.427	-0.275	-0.419
	tetrapyrrole biosynthesis I (from glutamate)	-0.599	-0.564	-0.739	-0.661	-0.699	-0.626
Fatty Acid and Lipid Biosynthesis	(5Z)-dodec-5-enoate biosynthesis	0.548	0.629	0.529	0.592	0.626	0.438
	mycolate biosynthesis	0.545	0.630	0.522	0.588	0.626	0.438
	oleate biosynthesis IV (anaerobic)	0.545	0.630	0.522	0.588	0.626	0.438
	palmitate biosynthesis II (bacteria and plants)	0.541	0.603	0.529	0.582	0.611	0.421
	palmitoleate biosynthesis I (from (5Z)-dodec-5-enoate)	0.548	0.629	0.529	0.592	0.626	0.438
	stearate biosynthesis II (bacteria and plants)	0.546	0.620	0.522	0.589	0.626	0.438
	superpathway of fatty acid biosynthesis initiation (E. coli)	0.542	0.628	0.516	0.582	0.626	0.438
Respiration	methanogenesis from acetate	0.501	0.409	0.345	0.130	0.163	0.498
TCA cycle	TCA cycle I (prokaryotic)	0.544	0.503	0.350	0.354	0.441	0.620
	TCA cycle V (2-oxoglutarate:ferredoxin oxidoreductase)	0.446	0.461	0.222	0.389	0.450	0.365
