# Supplementary material for: Synergistic anti-obesity effects of Bifidobacterium breve BR3 and Lactiplantibacillus plantarum LP3 via coordinated regulation of lipid metabolism and gut microbiota
Source: J Microbiol. 2025 Dec 31;63(12):e2511001. doi: 10.71150/jm.2511001 (PMC13577009; doi:10.71150/jm.2511001)
Supplement: Supplementary file 5 [file jm-2511001-Supplementary-Table-S2.pdf]

Table S2. Spearman correlation (*p*-value) between functional pathways and obesity biomarkers

Superclass	Functional pathway	*p*-value					
		DXA – fat mass	EchoMRI – fat mass	Total Cholesterol (mg/dL)	Glucose (mg/dL)	AST (U/L)	ALT (U/L)
Fermentation	acetylene degradation	0.016	0.007	0.036	0.009	0.006	0.095
	hexitol fermentation to lactate, formate, ethanol and acetate	0.031	0.013	0.023	0.009	0.067	0.186
	homolactic fermentation	0.013	0.004	0.027	0.003	0.002	0.057
	mixed acid fermentation	0.029	0.020	0.044	0.011	0.012	0.133
	pyruvate fermentation to acetate and lactate II	0.003	0.001	0.017	0.002	0.001	0.022
Cofactor, Prosthetic Group, Electron Carrier, and Vitamin Biosynthesis	1,4-dihydroxy-2-naphthoate biosynthesis I	0.009	0.018	0.015	0.007	0.027	0.003
	8-amino-7-oxononanoate biosynthesis I	0.001	0.000	0.003	0.001	0.000	0.013
	adenosylcobalamin biosynthesis from cobyrinate a,c-diamide I	0.011	0.004	0.011	0.002	0.002	0.056
	adenosylcobalamin salvage from cobinamide I	0.012	0.004	0.015	0.003	0.003	0.064
	adenosylcobalamin salvage from cobinamide II	0.009	0.003	0.009	0.001	0.001	0.045
	biotin biosynthesis I	0.001	0.000	0.003	0.001	0.000	0.011
	heme biosynthesis I (aerobic)	0.000	0.001	0.003	0.061	0.003	0.001
	superpathway of heme biosynthesis from glutamate	0.000	0.001	0.004	0.060	0.003	0.001
	superpathway of demethylmenaquinol-8 biosynthesis	0.009	0.019	0.015	0.007	0.027	0.003
	superpathway of menaquinol-11 biosynthesis	0.009	0.022	0.015	0.008	0.036	0.003
	superpathway of menaquinol-12 biosynthesis	0.009	0.022	0.015	0.008	0.036	0.003
	superpathway of menaquinol-13 biosynthesis	0.009	0.022	0.015	0.008	0.036	0.003
	superpathway of menaquinol-7 biosynthesis	0.013	0.030	0.020	0.011	0.042	0.003
	superpathway of menaquinol-8 biosynthesis I	0.009	0.021	0.015	0.010	0.041	0.002
	superpathway of phylloquinol biosynthesis	0.009	0.018	0.015	0.007	0.027	0.003
	superpathway of tetrahydrofolate biosynthesis	0.002	0.001	0.019	0.002	0.003	0.030
	superpathway of tetrahydrofolate biosynthesis and salvage	0.003	0.001	0.022	0.002	0.004	0.038
	coenzyme A biosynthesis I	0.002	0.001	0.012	0.002	0.003	0.051
	NAD salvage pathway II	0.007	0.004	0.019	0.019	0.108	0.026
	tetrapyrrole biosynthesis I (from glutamate)	0.001	0.002	0.000	0.000	0.000	0.001
Fatty Acid and Lipid Biosynthesis	(5Z)-dodec-5-enoate biosynthesis	0.003	0.001	0.004	0.001	0.001	0.021
	mycolate biosynthesis	0.003	0.000	0.004	0.001	0.001	0.021
	oleate biosynthesis IV (anaerobic)	0.003	0.000	0.004	0.001	0.001	0.021
	palmitate biosynthesis II (bacteria and plants)	0.003	0.001	0.004	0.001	0.001	0.025
	palmitoleate biosynthesis I (from (5Z)-dodec-5-enoate)	0.003	0.001	0.004	0.001	0.001	0.021
	stearate biosynthesis II (bacteria and plants)	0.003	0.001	0.004	0.001	0.001	0.021
	superpathway of fatty acid biosynthesis initiation (E. coli)	0.003	0.001	0.005	0.001	0.001	0.021
Respiration	methanogenesis from acetate	0.006	0.024	0.049	0.272	0.234	0.009
TCA cycle	TCA cycle I (prokaryotic)	0.003	0.006	0.047	0.045	0.020	0.001
	TCA cycle V (2-oxoglutarate:ferredoxin oxidoreductase)	0.014	0.012	0.149	0.030	0.018	0.048
