# Supplementary material for: Synergistic anti-obesity effects of Bifidobacterium breve BR3 and Lactiplantibacillus plantarum LP3 via coordinated regulation of lipid metabolism and gut microbiota
Source: J Microbiol. 2025 Dec 31;63(12):e2511001. doi: 10.71150/jm.2511001 (PMC13577009; doi:10.71150/jm.2511001)
Supplement: Supplementary file 6 [file jm-2511001-Supplementary-Table-S3.pdf]

Table S3. List of primers used in present study

Target		Primer sequence 5'-3'	References (DOI)
Adiponectin	F	AAG GAG ATG CAG GTC TTC TTG GT	10.1101/2023.05.16.540975
	R	CAC TGA ACG CTG AGC CAT ACA T	
Sirt1	F	CCA GAC CCT CAA GCC ATG TT	10.1016/j.jcmgh.2022.12.013
	R	CTG TCC GGG ATA TAT TTC CTT TGC	
Pparα	F	TAA TTT GCT GTG GAG ATC GGC	10.1016/j.aohep.2019.03.013
	R	GGT GTC ATC TGG ATG GTT GC	
Srebp-1c	F	ATG CCA TGG GCA AGT ACA CA	10.1002/biof.1885, 10.1194/jlr.M093252
	R	AGA TCT CTG CCA GTG TTG CC	
Fas	F	ACC ACT GCA TTG ACG GCC GG	10.1194/jlr.M081745
	R	GGG TCA GGC GGG AGA CCG AT	
Cebpα	F	TTA CAA CAG GCC AGG TTT CC	10.1016/j.fct.2011.11.020
	R	GGC TGG CGA CAT ACA GTA CA	
Pparγ	F	ATT GAG TGC CGA GTC TGT GG	10.1186/1478-811X-11-74
	R	GCC CAA ACC TGA TGG CAT TG	
Scd1	F	CAG GTT TCC AAG CGC AGT TC	10.4093/dmj.2017.0084
	R	GAA CTG GAG ATC TCT TGG AGC A	
Fabp4	F	AGA AGT GGG AGT GGG CTT TG	10.1038/s41598-022-07379-x
	R	CAC GCC CAG TTT GAA GGA AA	
Acc	F	GGG CAC AGA CCG TGG TAG TT	10.1172/JCI33594.
	R	CAG GAT CAG CTG GGA TAC TGA	
Tnf-α	F	GCC ACC ACG CTC TTC TGT CT	10.1371/journal.pone.0117120
	R	GGC TGA TGG TGT GGG TGA GG	
Mcp-1	F	AGG TCC CTG TCA TGC TTC TG	10.1186/s40885-019-0118-8
	R	TCT GGA CCC ATT CCT TCT TG	
Il-1β	F	AAA TAC CTG TGG CCT TGG GC	10.1074/jbc.RA117.001526
	R	CTT GGG ATC CAC ACT CTC CAG	
iNOS	F	AAT CTT GGA GCG AGT TGT GG	10.1016/j.jgr.2022.10.005
	R	CAG GAA GTA GGT GAG GGC TTG	
Cd11c	F	ACG TCA GTA CAA GGA GAT GTT GGA	10.1152/ajpendo.00082.2019
	R	ATC CTA TTG CAG AAT GCT TCT TTA CC	
Arginase1	F	ATG GAA GAG ACC TTC AGC TAC	10.1186/s13395-015-0048-4
	R	GCT GTC TTC CCA AGA GTT GGG	
Il-10	F	CAT GGC CCA GAA ATC AAG GA	10.1210/me.2007-0420
	R	GGA GAA ATC GAT GAC AGC GC	
Mgl1	F	ATG ATG TCT GCC AGA GAA CC	10.1155/2012/762094
	R	ATC ACA GAT TTC AGC AAC CTT A	
Zo-1	F	GCC GCT AAG AGC ACA GCA A	10.1186/s12885-016-2180-x
	R	CCC CAC TCT GAA AAT GAG GA	
Occludin	F	TAC TGG TCT CTA CGT GGA TCA AT	10.3389/fnmol.2020.00002
	R	TTC TTC GGG TTT TCA CAG CAA	

Claudin-2	F	TAT GTT GGT GCC AGC ATT GT	10.1038/srep32889
	R	TCA TGC CCA CCA GAG AGA TA	
Claudin-5	F	GCT CAG TGC ACC ACC TGC GT	10.1006/excr.2000.5113
	R	GAA CCA GCA GAG CGG CAC GA	
Claudin-8	F	CAT GCC AAC ATC AGA ATG CAG T	10.1038/s41598-017-12494-1
	R	CTG TGG TCC AGC CTA TGT AGA G	
Claudin-15	F	GCC TGT GGG ATG GTG GCT ATC TCG T	10.1038/s41598-017-12494-1
	R	TGG TGG CTG GTT CCT CCT TG	
Claudin-22	F	TTC CGA ACG GCAACGCAGGC	10.3390/nu12061862
	R	CCC ATC CCA GCA GGG AGA GCA	
